# Artificial shading of teinturier grape Kolor clusters reveals light-responsive biomarkers: focus on flavonoid profiles and metabolism

**DOI:** 10.3389/fpls.2024.1356799

**Published:** 2024-03-12

**Authors:** Huiqing Li, Xiaotong Gao, Yu Wang, Haocheng Lu, Mengbo Tian, Changqing Duan, Jun Wang

**Affiliations:** ^1^ Center for Viticulture and Enology, College of Food Science and Nutritional Engineering, China Agricultural University, Beijing, China; ^2^ Key Laboratory of Viticulture and Enology, Ministry of Agriculture and Rural Affairs, Beijing, China; ^3^ Key Laboratory of Agricultural Product Fine Processing and Resource Utilization, Ministry of Agriculture and Rural Affairs, Anhui Engineering Research Center for High Value Utilization of Characteristic Agricultural Products, College of Tea and Food Science and Technology, Anhui Agricultural University, Hefei, China

**Keywords:** microclimate, anthocyanins, flavonols, skin, pulp, light

## Abstract

Kolor is a teinturier grape cultivar, that accumulates flavonoids in the skin and pulp. However, the concentrations and proportions of flavonoids in Kolor skin and pulp differ, suggesting tissue specificity in teinturier grapes. Light conditions significantly influence the evolution of flavonoids. Moreover, studies on the mechanisms governing flavonoid accumulation in light response sensitivity of teinturier grapes are limited. In the three consecutive years of study, the exposure of Kolor clusters was altered by bagging from pre-veraison to harvest. QqQ/MS and RT‒qPCR wereused to determine the individual anthocyanin contents and the relative gene expression. There was a significant decrease in the total anthocyanins and flavonols in the Kolor berries, with flavonols showing greater sensitivity to bagging. Bagging did not exert a consistent impact on the flavan-3-ols in Kolor berries. The sensitivities of anthocyanins in Kolor skin and pulp differed under light exclusion conditions. The concentration of trihydroxy-substituted anthocyanins in the skin decreased, while the proportion of dihydroxy-substituted anthocyanins in the pulp significantly increased, but the anthocyanin concentration in the pulp did not change significantly after bagging. The contents of malvidins and quercetins in the skin, and myricetins and quercetins in the pulp, were significantly reduced after bagging. The expression of flavonoid synthesis genes in Kolor skin and pulp was tissue-specific. After bagging, *UFGT* expression increased in the pulp and decreased in the skin. In addition, *LDOX*, *FLS-1*, *CHI-1*, *CHI-2*, *F3H-1*, *F3H-2*, and *MYB4a* exhibited sensitive light responses in both the skin and pulp. This study offers new insights into the regulation of flavonoids in Kolor grapes under light exclusion conditions.

## 1 Introduction

Anthocyanins are crucial pigments in grapes. In typical red grape varieties, the skin is purple or red, while the pulp remains colourless. However, certain teinturier grape varieties accumulate anthocyanins in both the skin and the pulp (He et al., 2010). Compared to those of non-teinturier grape varieties, the anthocyanin profiles of several teinturier grapes were unique. It has been reported that peonidin-3,7-*O*-*β*-diglucoside was detected in Garnacha Tintorera (Castillo-Munoz et al., 2010), and pelargonidin-3-*O*-*β*-glucoside was found in Yan 73 (He et al., 2010). Research has shown that teinturier grapes have significantly greater anthocyanin concentrations than nonteinturier grape varieties (Tian et al., 2021). The profiles of anthocyanins in the pulp and skin of teinturier grapes are highly similar (Ageorges et al., 2006), but the proportions of certain anthocyanins exhibit tissue specificity. The skin contains more trihydroxy-substituted anthocyanins, while the pulp is richer in dihydroxy-substituted anthocyanins (Guan et al., 2019). In addition to anthocyanins, flavonols and flavan-3-ols are also synthesized in the pulp of teinturier grapes (Yue et al., 2018). Teinturier grapes, which have elevated flavonoid concentrations, are frequently used to modify the colour of red wines, elevate tannin levels, and increase ageing potential (Chen et al., 2018).

The accumulation of flavonoids in grapes is regulated by structural genes in the flavonoid pathway, such as chalcone synthase (*CHS*), chalcone isomerase (*CHI*), flavanone 3-hydroxylase (*F3H*), flavonoid-3’-hydroxylase (*F3’H*), flavonoid-3’5’-hydroxylase (*F3’5’H*), leucoanthocyanidin dioxygenase (*LDOX*), and UDP-glucose: flavonoid 3-*O*-glucosyltransferase (*UFGT*) (Xie et al., 2019; Wei et al., 2023). In addition, the biosynthesis of anthocyanins is intricately regulated by transcription factors. Plant studies suggest that anthocyanin biosynthesis primarily involves three transcription factor families: *MYB*, *bHLH*, and *WD40* (Hichri et al., 2010). Typically, the MBW transcriptional complex (*MYB*-*bHLH*-*WD40*) is formed, and these factors regulate the expression of structural genes, thus governing anthocyanin synthesis (Xu et al., 2015). Flavonoid compounds in plants are not solely under genetic control; they are also subject to external environmental factors such as light, temperature, and moisture (Sun et al., 2019). Among these factors, light is particularly pivotal, because it influences the ripening of grape berries and the metabolism of flavour compounds. ELONGATED HYPOCOTYL 5 (*HY5*) actively participates in regulating light morphogenesis and anthocyanin synthesis in plants (Loyola et al., 2016). Serving as a crucial light signalling element, *HY5* activates the expression of genes associated with the anthocyanin biosynthetic pathway, especially *CHS*, *DFR*, and *LDOX*, under light conditions (Gollop et al., 2002).

In contrast to certain fruits, which are dependent on ample light, grapes can accumulate flavonoids, including anthocyanins, in their skin under various light conditions (Koyama et al., 2012). Nevertheless, variations in the accumulation of flavonoids can arise due to differences in light intensity and quality (Matus et al., 2009). In the context of wine grapes, extensive research has been conducted on the shading of grape clusters and its influence on anthocyanin accumulation in grape berries. Artificial shading typically employs cover boxes, curtains, or bags to impose light-deprivation conditions on grape clusters (Ristic et al., 2007; Downey et al., 2008; Koyama and Goto-Yamamoto, 2008). The results from certain shading experiments revealed that, following shading treatment during the ripening stage, there was a significant reduction in anthocyanin content in the grape skin, accompanied by a marked decrease in the expression of genes associated with anthocyanin synthesis (Koyama and Goto-Yamamoto, 2008; Li et al., 2013). Conversely, when shading treatment was administered in the early stages of cluster development, there was no significant alteration in the overall anthocyanin content in mature berries; however, there was an increase in the proportion of 3’-hydroxylated anthocyanins in the grape skin (Ristic et al., 2007; Downey et al., 2008). The impact of shading treatments on the concentration and proportion of anthocyanins in berries varies at specific developmental stages (Dokoozlian and Kliewer, 1996).

Presently, extensive research concerning the flavonoid pathway in nonteinturier grape varieties under light exposure and exclusion conditions is underway. However, the influence of light intensity on fruit quality in teinturier grapes remains unclear. Experimental findings indicate that shading treatments applied to clusters of teinturier grapes result in reduced amino acid concentrations in both the skin and pulp, a decreased proline proportion, and an increased GABA proportion (Guan et al., 2017). Additionally, shading significantly diminishes the levels of sugar, ABA, and anthocyanins in coloured grape fruits, altering the ratio of dihydroxylated to trihydroxylated anthocyanins. Notably, these alterations are more pronounced in the skin than in the pulp (Guan et al., 2016). A reduction in the anthocyanin concentration in teinturier grapes following shading correlated with decreases in the expression of *UFGT*, *MybA1*, *MybA2*, and *Myc1*. Similarly, changes in the dihydroxylated to trihydroxylated anthocyanin ratio are linked to the expression levels of *F3’H* and *F3’5’H* (Guan et al., 2014). Experiments on light recovery after shading in teinturier grapes revealed that, in comparison to those in shaded berries, there were increases in the expression levels of light-responsive factors, transcription factors, and structural genes involved in the anthocyanin synthesis pathway in recovered berries. This highlighted the pivotal role of light exposure in the production of anthocyanins and other biological transformations in teinturier grapes (Li et al., 2023). To date, research on flavonoids in teinturier grapes has focused mainly on anthocyanins, and studies on the profiles of other flavonoid compounds are very limited. However, investigations into the light-responsive specificity of genes related to the flavonoid biosynthesis pathway in the skin and pulp of teinturier grapes have not been comprehensive enough.

The grape cultivar Kolor (*Vitis vinifera* L.) was developed at the Freiburg Research Institute in Germany through a crossbreeding of Pinot Noir and Teinturier. Specifically, bred for the production of coloured grape wine resembling that of pigmented grapes, Kolor was introduced to China from New Zealand in 2011 (Chen et al., 2018). Research on the flavonoid profiles of Kolor berries is limited. Bagging, an environmentally friendly method commonly employed in table grape cultivation, is utilized to modify light conditions for berries. It serves to shield grapes from sunlight, diseases, insects, and birds, preventing berries from cracking and reducing chemical residues. This method also contributes to delaying berry ripening (Karajeh, 2018). In this study, fruit bags were applied to modify the light intensity to which the teinturier grape cultivar Kolor was exposed from the pre-veraison to harvest stages. Our objective was to investigate the influence of bagging treatment on the flavonoid profiles of Kolor berries and the expression levels of flavonoid biosynthesis-related genes. Furthermore, the differential effects of bagging on the skin and pulp of Kolor were also discussed. This study offers new insights into the regulation of flavonoids in Kolor grapes under light exclusion conditions.

## 2 Materials and methods

### 2.1 Chemicals

Acetonitrile, formic acid, and methanol (HPLC grade) were supplied from Fisher (Fairlawn, NJ, USA). The standards (purities > 95%) used for identification and quantification of flavonoids: (+)-catechin (Ca), (-)-epicatechin (EC), (-)-epigallocatechin (EGC), (-)-gallocatechin (GC) and (-)-epicatechin-3-*O*-galate (ECG), quercetin-3-*O*-glucoside, and malvidin-3-*O*-glucoside were purchased from Extrasynthese (France), myricetin-3-*O*-galactoside, kaempferol-3-*O*-galactoside, kaempferol-3-*O*-glucoside, syringetin-3-*O*-glucoside, isorhamnetin-3-*O*-glucoside were purchased from Push Bio-technology (Chengdu, China). Other analytical grade chemical reagents, such as phloroglucinol, sodium acetate, ascorbic acid, NaOH, HCl, methanol, acetone, were supplied from Macklin Biochemical (Shanghai, China).

### 2.2 Vineyard and vine management

The experiments were carried out over three consecutive growing seasons (2018 to 2020) on Kolor vines (Simple sequence repeat [SSR] identification and comparison results of Kolor are shown in **Supplementary Table 1**) at Shangzhuang Experimental Station of China Agricultural University in Haidian district, Beijing, China (40°14′ N, 116°19′ E, at an altitude of 50 m; **Supplementary Figure 1B**). The vines, which were planted in 2014, were organized in north-south rows with interrow and intrarow vine spacings of 2.5 m × 1.2 m. Grapevines were trained using a modified vertical shoot positioning (M-VSP) trellis system and maintained at 12-15 nodes per linear metre (Cheng et al., 2014). The canopy was approximately 120 cm tall and approximately 70 cm thick. The soil type in the vineyard is classified as ‘loam’ (Cheng et al., 2020). All aspects of vineyard management, including drip irrigation, nutrition, and pest control, adhere to standardized practices. Furthermore, meteorological data, including mean monthly temperature (°C), sunshine duration (h), and rainfall (mm) during the growing season, were sourced from the China Meteorological Data Sharing Service System (http://cdc.cma.gov.cn/).

### 2.3 Experimental design and sampling

Individual grape clusters were enclosed in two-layer Kraft paper bags (38 cm × 26 cm) with yellow exteriors and black interiors, coated with wax. To ensure ventilation, straw was placed inside the bags (**Supplementary Figure 1A**). The bagging treatment commenced immediately following the sampling of E-L 33 and continued for a duration of two months until harvest, while untreated grapes served as the control. Each treatment was applied to 90 randomly selected clusters from both sites of the canopy, with three biological replicates. As part of this treatment, a microclimate weather station was installed to meticulously monitor the microclimate in the grape cluster area. Positioned beneath the canopy of the grape clusters, the microclimate station was enveloped with bags identical to those used for the grape clusters, enabling the detection of microclimate changes within the bags. An identical weather station, situated at the same elevation but without bags, served as the control group. To monitor the vineyard’s microclimate, we installed solar radiation (SR) sensors (S-LIB-M003, Onset, USA), photosynthetically active radiation (PAR) sensors (S-LIA-M003, Onset, USA), and temperature-humidity sensors (S-THB-M002, Onset, USA). The sensor probes were positioned with their heads pointing upwards. Meteorological data were recorded at 15-minute intervals using a HOBO micro station (H21-002, Onset, USA). Microclimatic conditions for both bagged and control grapes were monitored following established procedures.

Healthy Kolor berries from each treatment were sampled at the following stages: E-L 33 (green berries), E-L 35 (veraison initiation), E-L 36 (medium maturity), E-L 37 (berries not fully ripe), and E-L 38 (harvest) (Coombe, 1995). The harvest date was determined at the technological maturity stage, characterized by a total soluble solid (TSS) content of more than 17°Brix and the absence of shrivelling. All the sampling dates are shown in **Supplementary Table 2**. Photos of Kolor berries are shown in **Supplementary Figure 1C**. For each replicate, 200 berries were randomly selected from 5-8 clusters at each sampling point. One hundred randomly selected berries from each replicate were weighed, juiced, and subsequently subjected to low-speed centrifugation. The supernatant was analysed for total soluble solids, pH, and titratable acid (TA) content according to the methods of by Wang et al. (2019). The remaining samples were promptly frozen in liquid nitrogen and stored at -80°C for subsequent metabolite and gene expression determination.

### 2.4 Extraction and QqQ/MS analysis of flavonoids in Kolor berries

Seventy frozen berries were randomly selected from each biological replicate. The skins and pulps were quickly separated, the seeds were extracted while frozen, and subsequently ground into powder using an IKA A11 basic analytical mill (Germany) in a liquid nitrogen environment. Following lyophilization under vacuum freeze-drying in LGJ-10 (China), the resulting dry powder was stored for extraction. The extraction of flavonoids followed a slight modification of the method by Chen et al. (2018). The steps for extracting anthocyanins and flavonols were as follows: weighed dry powder (0.100 g) was immersed in 1.0 mL of a 50% methanol aqueous solution, subjected to low-temperature ultrasound sonication for 20 minutes, and then centrifuged at 13300 × g at 4°C for 10 minutes. The residues underwent a second extraction. The steps for extracting free flavan-3-ols were as follows: 0.100 g of dry powder was mixed with 1.0 mL of a 70% acetone aqueous solution containing 0.5% ascorbic acid. The mixture was agitated for 15 minutes and subsequently centrifuged at 5910 × g at 4°C for 15 minutes. The residue was subjected to a second extraction. The supernatant was evaporated under nitrogen until completely dry and then reconstituted with an equal volume of a 1% hydrochloric acid methanol solution/200 mM sodium acetate aqueous solution (1:1, v/v). The extraction of proanthocyanins proceeded as follows: 0.100 g of dry powder was mixed with 1.0 mL of a phloroglucinol buffer (50 g/L phloroglucinol, 0.3 M HCl, and 5 g/L ascorbic acid dissolved in methanol). After the mixture was incubated at 50°C for 20 minutes, 1.0 mL of a 200mM sodium acetate aqueous solution was added, followed by centrifugation for 15 minutes at 5910 × g. The residue underwent two additional extractions. The supernatants were collected and the residue was re-extracted twice. All the supernatants were collected and stored at -40°C for subsequent analysis using QqQ/MS.

Flavonoids were separated using an Agilent 1200 series HPLC-MSD trap VL system, featuring a Poroshell 120 EC-C18 column (150 × 2.1 mm, 2.7 μm), and coupled with an Agilent 6410 triple-quadrupole (QqQ) instrument equipped with an electrospray ionization (ESI) source, with some parameters modified based on Li et al. (2016). Data processing was conducted using Agilent MassHunter Qualitative Analysis software version B.10.00. The product contents were determined using external standards under identical HPLC conditions. Flavan-3-ols were quantified using (+)-catechin, (-)-epicatechin, (-)-epigallocatechin, (-)-gallocatechin and (-)-epicatechin-3-*O*-galate, while anthocyanins were quantified using malvidin-3-*O*-glucoside. For flavonols, quantification was based on myricetin-3-*O*-galactoside, quercetin-3-*O*-glucoside, kaempferol-3-*O*-galactoside, kaempferol-3-*O*-glucoside, syringetin-3-*O*-glucoside, and isorhamnetin-3-*O*-glucoside.

### 2.5 Colorimetric measurements of the extraction

The extraction method mirrored that used for anthocyanins, and all the measurements were conducted in triplicate for consistency. The colour parameters were determined after filtration through a 0.22 μm organic membrane filter. For each sample, 100 μL of filtered liquid was added. Chromatic characteristics were assessed using a Spectra Max 190 spectrophotometer (Molecular Devices, USA) by recording UV-Visible spectra from 400 to 700 nm with a 1 nm bandwidth, and employing a 50% methanol aqueous solution as a reference. The colour parameters of the extracted liquid, which included L* (lightness), a* (red-green component), b* (yellow-blue component), C* (chroma), *h* (hue angle), and *ΔE* (color difference), were assessed using the CIELAB coordinates (Zhang et al., 2015). C*, *h*, and *ΔE* were calculated using the following equations: C* = *(a∗* ^2^ + *b∗* ^2^)^1/2^, *h*= tan-1 (b*/a*) and *ΔE*=[(ΔL*)^2^+(Δa*)^2^+(Δb*)^2^]^1/2^.

### 2.6 RNA extraction, cDNA synthesis, and RT-qPCR analysis

The skin and pulp were separated from Kolor grapes in the 2018 season and ground into powder before extraction. RNA extraction was carried out according to the instructions provided with the RNAprep Pure Plant Kit (DP432, TIANGEN, China). For reverse transcription, 1 µg of total RNA was used with a Vazyme kit (R333, China). RT-qPCR was conducted using SYBR master mix (Q711, Vazyme, China) on Bio-Rad CFX384 instrument (USA). UBIQUITIN served as the reference gene. A total of fourteen structural genes, five MYB family transcription factors closely associated with the flavonoid biosynthesis pathway, and two light-regulated factors, *HYH* and *HY5*, were selected for investigation. The sequences of primers used are provided in **Supplementary Table 3**. Each reaction was performed in triplicate, and the results were analysed using Bio-Rad’s CFX Maestro Software (USA).

### 2.7 Statistical analysis

Differences in the means of the concentrations of the compounds were determined via ANOVA with Duncan’s multiple range test at a level of *p* < 0.05 using the ‘agricolae’ package in the R statistical environment (4.1.2). Unsupervised principal component analysis (PCA) and orthogonal partial least squares discriminant analysis (OPLS-DA) of the metabolic profiles were performed using SIMCA v14.1 software (Umetrics, Umea, Sweden). Network visualization was performed using Gephi software (France). Graphs were prepared using Excel 2019. The results are presented as the mean ± SD of three biological replicates. All the concentrations of flavonoids are expressed as mg/kg berry fresh weight (mg/kg FW) in this study.

## 3 Results

### 3.1 Monitoring the meteorological conditions, microclimate and physicochemical parameters of the Kolor berries

The vineyard in the present study is located in Beijing, which has a continental monsoon climate (shown in **Supplementary Figure 2**; **Supplementary Table 4**). In 2018, the total precipitation during the entire growing season was the highest, while it was the lowest in 2019. Precipitation in 2018 was predominantly concentrated during the veraison stage of the grape growing season, accumulating to a total of 266.5 mm, surpassing that in 2019 (62.7 mm) by more than fourfold and exceeding that in 2020 (26.6 mm) by more than tenfold. Greater precipitation led to a comparatively extended veraison period for Kolor berries in 2018 than in the other two years. Notably, in 2018, the growing degree days (GDD) during the growing season exceeded 1630.8°C, notably surpassing the values observed in the other two years, especially during the ripening stage, where it reached 447.1°C. In contrast, in 2019 and 2020, the GDD registered values were 1471.5°C and 1447.4°C, respectively. Among the three seasons, vintage 2018 experienced higher temperatures and more rainfall, resulting in a relatively extended grape growing season. In contrast, 2019 had the lowest precipitation over the three-year period, along with the lowest relative air humidity, making it relatively drier than both 2018 and 2020 were.

**Supplementary Figure 3** reveals that the bags provided complete shielding from both the SR and PAR, ensuring that no light penetrated the bags. In the bags, the temperature was consistently maintained between 20.3°C and 33.7°C, and the humidity ranged from 35.3% to 100%. External to the bags, the temperature fluctuated between 19.8°C and 31.9°C, with relative humidity ranging from 38.4% to 99.9%. The temperature and humidity differences between the interior and exterior of the bags ranged from 0°C to 3.2°C and from 0.1% to 7.6%, respectively. Despite bagging treatment causing some variations in the temperature and humidity parameters the of grape clusters, the temperature inside the bags consistently remained below 35°C. This would prevent anthocyanin degradation, ensuring that the temperature is within the optimal range for anthocyanin biosynthesis in grape berries (Mori et al., 2005). In summary, these findings suggest that bagging treatments effectively block sunlight exposure without significantly affecting other microclimate indicators of grape clusters.

**Figure 1:**
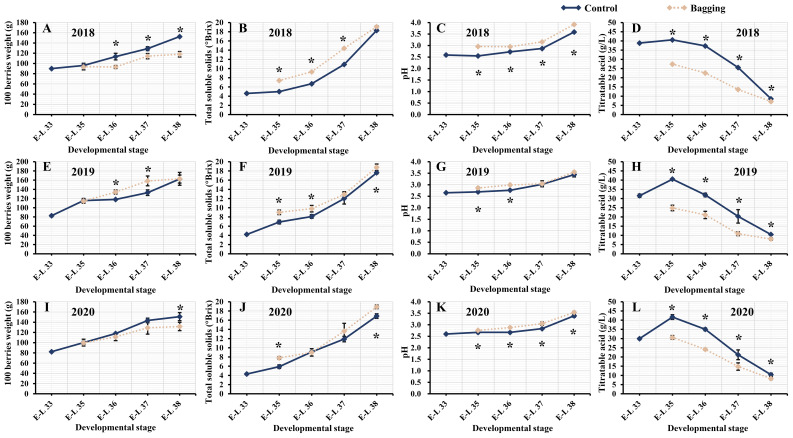
Physicochemical parameters of Kolor berries in five development stages from 2018 to 2020, 100 berries weight **(A, E, I)**, total soluble solids **(B, F, J)**, pH **(C, G, K)**, titratable acid **(D, H, L)**. The dark blue line represents the control groups and the yellow line represents the bagging treatment groups. * indicates there are significant differences between bagging and control groups (*p* < 0.05, *t*-test).

Throughout three consecutive experimental seasons of berry development, both the bagged and control groups exhibited analogous trends in the physicochemical parameters of the Kolor berries. The weight, TSS content, and pH of the 100 grapes gradually increased during berry development. The TA content in all Kolor berries reached its peak at the E-L 35 stage, and subsequently decreased as the berries matured. **Figure 1** shows a notable reduction in the weight of 100 mature berries after bagging in 2018 and 2020, while there was no significant change in 2019. Due to the postponed veraison of bagged berries compared to those outside the bags, bagged berries exhibited greater TSS during veraison. Nevertheless, in 2018, there were no significant differences in TSS among berries sampled at E-L 38, with only 2019 and 2020 indicating significantly greater TSS in the bagged treatment group than in the control group. The TSS of the mature berries, both inside and outside the bags, exceeded 17°Brix. After bagging, the TA content of the berries significantly decreased during development. From the onset of veraison until maturity, the difference in TA content between the bagged and control berries gradually decreased, and the difference in TA content during the mature stage was approximately 2 g/L.

### 3.2 Influence of bagging treatment on the composition and proportion of flavonoids in Kolor berries

In the present study, a total of 20 monomeric anthocyanins were detected in the skin (S) and pulp (P) of Kolor grape berries. These anthocyanins were classified into five main categories: delphinidins, cyanidins, petunidins, peonidins, and malvidins. As shown in **Supplementary Table 5**, there were five nonacylated anthocyanins, five acetylated anthocyanins, five caffeoylated anthocyanins and five coumaroylated anthocyanins. A total of 15 flavonols were detected (shown in **Supplementary Table 6**), including five quercetins, three kaempferols, three myricetins, two isorhamnetins, and one each of laricitrin and syringetin. Free flavan-3-ols and proanthocyanidins were detected in both the skin and pulp of Kolor berries, with a total of six kinds of free flavan-3-ols (Ca, EC, GC, EGC, ECG and EGCG), four types of extension subunits (Ca, EC, EGC and ECG) and five types of terminal subunits (Ca, EC, GC, EGC and ECG) was shown in **Supplementary Table 7**.

**Figure 2:**
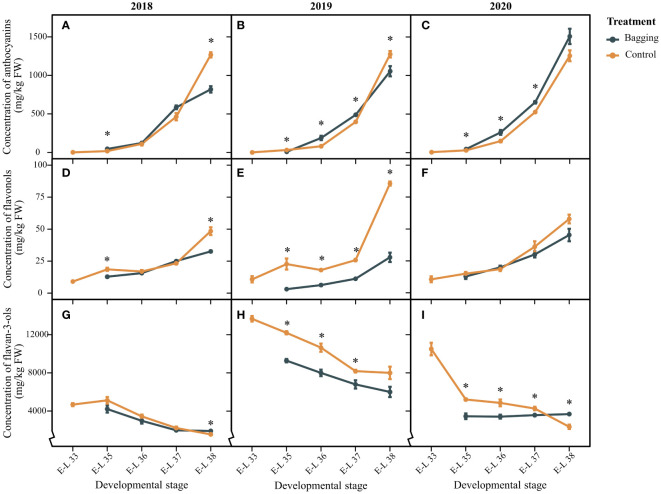
Total anthocyanins **(A-C)**, flavonols **(D-F)**, and flavan-3-ols **(G-I)** concentration in the Kolor berries over development stages under bagging treatment and control. The dark blue line represents the control groups and the yellow line represents the bagging treatment groups. * indicates there are significant differences between bagging and control groups (*p* < 0.05, *t*-test).

**Figure 2** shows the variations in the accumulation of anthocyanins, flavanols, and flavan-3-ols in Kolor berries from 2018 to 2020. Over three vintages, the anthocyanin concentration remained consistently stable in the control group, ranging from 1265.69 to 1275.03 mg/kg. Influenced by bagging treatment, the level of anthocyanins in mature berries decreased significantly to 818.66 mg/kg and 1054.35 mg/kg in 2018 and 2019, respectively, with no significant difference in 2020. Unlike those of anthocyanins, there was marked variability in flavonol concentrations in Kolor berries among different years. The flavonol concentration in the control group ranged from 48.39 to 85.60 mg/kg, with the lowest occurring in 2018 and the highest occurring in 2019. Like for anthocyanins, bagging exhibited a comparable pattern of flavonol dynamics. With the exception of 2020, bagging led to a substantial reduction in flavonol concentrations in mature berries, amounting to decreases of 32.8% and 67.3%, respectively. The flavan-3-ol concentration in Kolor berries showed notable variations among the three years. In particular, the flavan-3-ol concentration peaked in 2019, reaching 6004.23 mg/kg in mature berries. Conversely, the concentrations were significantly lower in 2018 and 2020, measuring 1553.76 mg/kg and 2352.68 mg/kg, respectively. Bagging resulted in a noteworthy increase in flavan-3-ol concentrations in mature berries in both 2018 and 2020. Nevertheless, during the developmental stages preceding maturity in 2019 and 2020, the flavan-3-ol concentration in the bagged berries was notably lower than that in the control group. Bagging not only impacts the flavonoid concentration in Kolor berries but also influences the proportion of flavonoids in mature berries (shown in **Supplementary Figure 4**). In bagged berries, the percentage of Mv anthocyanins significantly decreased, decreasing by 62%, 26%, and 25% in the corresponding years. Conversely, the proportion of Pn anthocyanins significantly increased after shading, with respective increases of 45%, 33%, and 18% in the three years. The Mv and Pn anthocyanins fall into the categories of dihydroxylated and trihydroxylated anthocyanins. The changes in the proportions of dihydroxylated and trihydroxylated anthocyanins in the bagged berries corresponded with Pn and Mv. The proportion of flavonols exhibited variations after bagging in different years, whereas the proportional changes in flavan-3-ols were not statistically significant. Notably, only the anthocyanin proportion demonstrated a consistent pattern in the bagging treatment group across different years.

### 3.3 Variation in light-responsive flavonoids in Kolor grape skin and pulp

**Figure 3:**
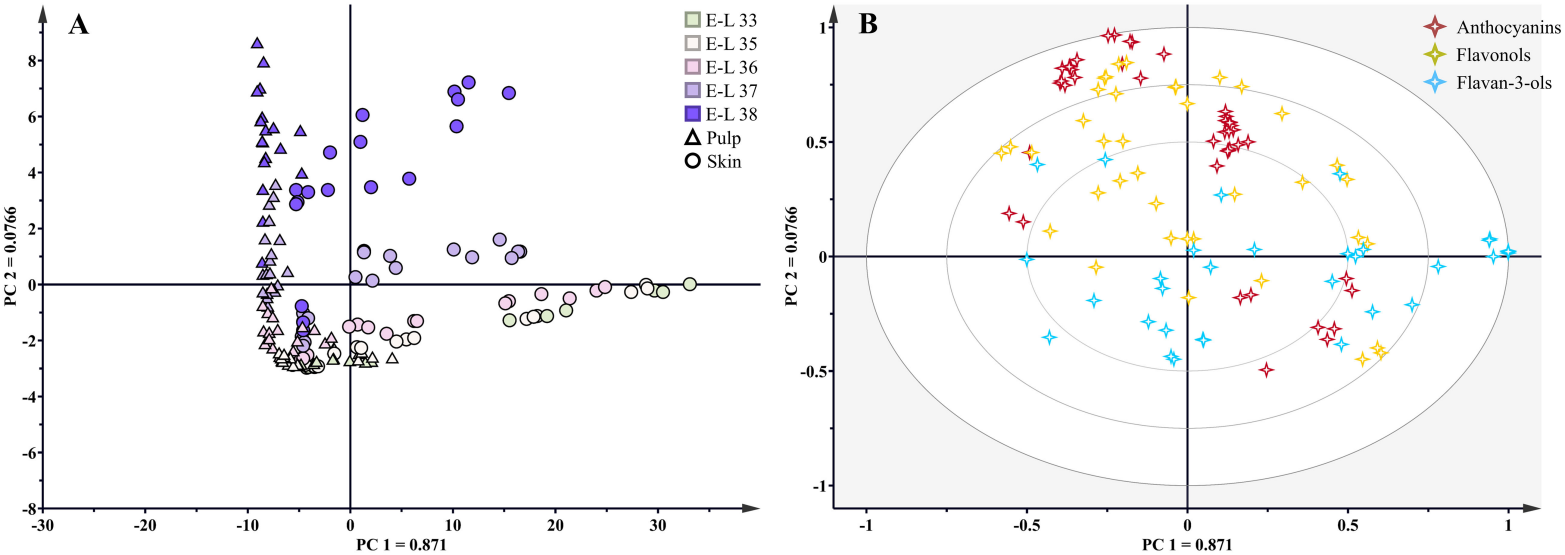
Principal component analysis **(A)** scores of flavonoids and distribution of all samples. Biplot **(B)** for S-C, S-B, P-C and P-B (S=Skin, P=Pulp, C=Control, B=bagging) at five developmental stages, different shapes represented different tissues, ‘△’ represented pulp and ‘○’ represented skin. ‘⋄‘ represented flavonoid compounds, different colors represented different kinds of flavonoids.

As a teinturier grape variety, the skin and pulp of Kolor berries display a vibrant red hue, with flavonoids present in both tissues. Throughout the growing season, the skin and pulp of Kolor berries exhibited similar flavonoid profiles, yet notable variations emerged in terms of concentration and proportion. PCA based on tissues and developmental stages (shown in **Figure 3**), revealed substantial variations in the concentration and proportion of flavonoids in Kolor berries. PC1 explained 87.1% of the variance and proficiently discriminated samples from distinct developmental stages, with samples exhibiting a clockwise rotation from the positive half-axis of the x-axis back to the first quadrant as the berry developed. Flavan-3-ols exhibited higher concentrations before veraison, with a gradual decline as the berry matured, whereas the concentrations of anthocyanins and flavonols exhibited the opposite pattern, peaking during the harvest period.

In the control group, compared with those of whole berries, the skin of mature berries contained 45%-56% anthocyanins, 54%-55% flavonols, and 76%-83% flavan-3-ols. In addition to the abundance of flavan-3-ols in the skin, both anthocyanins and flavonols also accumulate abundantly in the pulp. While the overall flavonoid profile in the Kolor berries remained stable after bagging, notable variations were observed in the concentrations and proportions of specific flavonoids. Over the course of the continuous three-year study, two primary factors, bagging treatment and year-to-year climate variation, influenced the flavonoids within Kolor berries. Using a two-way ANOVA, we determined flavonoids that exhibited significant differences after bagging. As shown in **Table 1**, the concentrations of most flavonoids in the skin significantly decreased after bagging. Specifically, the concentration of anthocyanins decreased from 632.82 mg/kg to 479.15 mg/kg, and the total flavonol concentration decreased from 35.19 mg/kg to 19.38 mg/kg. The total flavanol content remained relatively stable in the bagged berries, except for a significant decrease in the concentration of free flavanols from 21.00 mg/kg to 15.75 mg/kg. Following bagging treatment, only the concentrations of Pn, F3’H-anthocyanins, and ECG in Kolor skin significantly increased. Furthermore, the mean degree of polymerization (mDP) of proanthocyanidins in Kolor skin significantly increased after bagging treatment. This suggested that bagging treatment can modify the structure of flavan-3-ols in the skin, leading to a reduction in the quantity of free molecules and increasing their presence in a polymeric state in grape skin. In contrast, fewer compounds in the pulp exhibited substantial concentration changes in response to bagging treatment compared to those in the skin. Some anthocyanins and their derivatives in the pulp displayed lower sensitivity to variations in light conditions than did those in the skin. Importantly, while there was no significant change in the total concentration of anthocyanins in the pulp, the concentration of anthocyanins in the F3’5’H pathway significantly decreased from 48.95 mg/kg to 30.23 mg/kg. Similarly, all flavanols underwent a considerable reduction, decreasing from 28.74 mg/kg to 15.87 mg/kg. The concentration of flavanols in the pulp significantly decreased, dropping from 746.79 mg/kg to 415.48 mg/kg.

**Table 1:** The concentration and significance analysis of flavonoids in Kolor skin and pulp from 2018 to 2020 in bagging and control groups.

Compounds (mg/kg FW)	Treatment		Significant			Treatment		Significant		
	S-B*	S-C	Year	Treatment	Year & Treatment	P-B	P-C	Year	Treatment	Year & Treatment
Cyanidins	7.28 ± 3.56	10.01 ± 2.63		*		41.9 ± 13.58	49.42 ± 10.49	**	*	
Delphinidins	6.37 ± 2.41	15.49 ± 6.2		**		0.41 ± 0.19	1.4 ± 0.87	**	**	**
Peonidins	191.44 ± 124.02	127.1 ± 41.61	**	**	**	575.37 ± 187.18	534.34 ± 105.12	**		*
Petunidins	14.13 ± 5.14	27.12 ± 6.8		**		1.64 ± 0.78	4.07 ± 2.21	**	**	**
Malvidins	259.94 ± 130.32	453.1 ± 63.19	**	**	**	28.18 ± 17.09	43.48 ± 15.78		**	**
F3’H-anthocyanins	198.72 ± 127.46	137.11 ± 42.57	**	**	**	617.27 ± 199.89	583.76 ± 115.38	**		*
F3’5’H-anthocyanins	280.44 ± 136.72	495.71 ± 71.82	**	**	**	30.23 ± 17.92	48.95 ± 18.83		**	**
total-anthocyanins	479.15 ± 260.67	632.82 ± 96.74	**	**	**	647.51 ± 214.19	632.71 ± 107.28	**		**
Myricetins	7.71 ± 2.85	11.26 ± 2.93	**	**	*	7.9 ± 2.09	15.06 ± 5.3	**	**	**
Laricitrins	1.15 ± 0.47	1.59 ± 0.53	**	**		0.44 ± 0.29	0.73 ± 0.32	*	*	
Quercetins	7.53 ± 3.93	18.45 ± 7.22	**	**	**	3.87 ± 1.09	7.32 ± 2.79	**	**	**
Isorlrhamnetins	0.81 ± 0.39	0.83 ± 0.29	**		*	1.27 ± 0.5	1.74 ± 1			
Kaempferols	1.02 ± 0.94	1.59 ± 1.09	**	*		2.07 ± 1.82	3.01 ± 1.69	**		
Syringetins	1.17 ± 0.42	1.47 ± 0.21	**	**	*	0.33 ± 0.2	0.88 ± 0.47	**	**	**
F3H-flavonols	1.02 ± 0.94	1.59 ± 1.09	**	*		2.07 ± 1.82	3.01 ± 1.69	**		
F3’5’H-flavonols	10.03 ± 3.69	14.32 ± 3.56	**	**	*	8.66 ± 2.35	16.67 ± 5.74	**	**	**
F3’H-flavonols	8.34 ± 4.17	19.28 ± 7.47	**	**	**	5.14 ± 1.43	9.06 ± 3.39	**	**	**
total-flavonols	19.38 ± 8.2	35.19 ± 10.46	**	**	**	15.87 ± 4.84	28.74 ± 7.88		**	**
Catechin	101.22 ± 45.65	95.7 ± 45.07	**		**	111.15 ± 43.89	259.25 ± 243.53	**	**	**
Epicatechin	3148.71 ± 1852.53	2894.38 ± 2366.21	**		*	290.25 ± 160.04	465.94 ± 336.49	*	*	**
Gallocatechin	6.42 ± 2	10.16 ± 2.35		**	**	0.31 ± 0.1	0.91 ± 0.55	**	**	**
Epigallocatechin	166.46 ± 84.69	200.97 ± 149.17	**	*	**	3.9 ± 1.29	9.24 ± 4.8	**	**	**
Epicatechin gallate	26.72 ± 26.44	20.44 ± 23.73	**	**	**	9.69 ± 10.17	11.16 ± 8.63	**		*
Epigallocatechin gallate	0.48 ± 0.23	0.5 ± 0.33	**		**	0.17 ± 0.12	0.23 ± 0.17			
F3’H-flavan-3-ols	3276.65 ± 1868.46	3010.53 ± 2394.91	**		*	411.09 ± 146.7	736.35 ± 576.74	**	**	**
F3’5’H-flavan-3-ols	173.36 ± 84.33	211.63 ± 150.74	**	*	**	4.39 ± 1.46	10.38 ± 5.43	**	**	**
total-free-flavan-3-ols	15.75 ± 3.48	21.00 ± 3.29	**	**		15.3 ± 5.42	13.79 ± 2.56	*		
total-flavan-3-ols	3450.01 ± 1947.44	3222.16 ± 2545.05	**		*	415.48 ± 148.03	746.79 ± 582.21	**	**	**
mDP	25.44 ± 9.21	20.49 ± 8.1	**	**		16.07 ± 6.73	15.41 ± 4.39	**		

* S, Skin; P, Pulp; C, Control; B, bagging, *represents p < 0.05, **represents p < 0.01.

Values were reported as means ± SD. Significance results consisted of two-way ANOVA.

In addition to light affecting the concentration of flavonoids, **Table 2** illustrates that the proportion of flavonoids exhibited diverse light response characteristics. Bagging treatment resulted in alterations in the ratios of anthocyanins in the F3’5’H and F3’H pathways of the skin. Specifically, the former decreased significantly in bagged berries, while the latter increased, aligning with the observed pattern in the pulp. Changes in the proportions of flavonols within the F3’5’H and F3’H pathways in the skin contrast those of anthocyanins. There were no significant differences in the proportions of various flavonols in the pulp in response to light following bagging. The differences in the proportions of flavanols in the skin and pulp were consistent, with GC and EGC being the only exceptions. Bagging treatment reduced their proportions in both the skin and pulp. Additionally, notable changes occurred in the proportions of flavanols within the F3’H and F3’5’H pathways, resulting in increased and decreased ratios, respectively, following bagging treatment.

**Table 2:** The proportions and significance analysis of flavonoids in Kolor skin and pulp from 2018 to 2020 in bagging and control groups.

Compounds (%)	Treatment		Significant			Treatment		Significant		
	S-B*	S-C	Year	Treatment	Year & Treatment	P-B	P-C	Year	Treatment	Year & Treatment
Cyanidins	1.68 ± 0.4	1.59 ± 0.4	*			6.51 ± 0.74	7.76 ± 0.56	*	**	*
Delphinidins	1.79 ± 1.08	2.44 ± 0.96	**			0.06 ± 0.02	0.24 ± 0.17		**	
Peonidins	35.94 ± 9.46	19.87 ± 5.32	**	**		89 ± 1.01	84.12 ± 3.46		**	
Petunidins	3.77 ± 1.89	4.27 ± 0.87	**		*	0.25 ± 0.05	0.68 ± 0.42		**	
Malvidins	56.83 ± 6.42	71.84 ± 4.63	**	**		4.17 ± 1.39	7.21 ± 3.33		*	*
F3’H-anthocyanins	37.61 ± 9.13	21.45 ± 5.34	**	**		95.51 ± 1.43	91.88 ± 3.91		*	
F3’5’H-anthocyanins	62.39 ± 9.13	78.55 ± 5.34	**	**		4.49 ± 1.43	8.12 ± 3.91		*	
Myricetins	40.63 ± 6.32	32.49 ± 3.78		**		50.78 ± 5.42	51.49 ± 4.54	*		
Laricitrins	6.07 ± 1.79	4.54 ± 0.85		*		2.52 ± 1.25	2.74 ± 1.23	*		
Quercetins	38.12 ± 7.73	51.27 ± 7.52		**		24.71 ± 2.49	24.88 ± 2.7			
Isorlrhamnetins	4.17 ± 1.49	2.37 ± 0.39		**		8.5 ± 3.2	6.03 ± 2.3			
Kaempferols	4.78 ± 3.35	4.88 ± 3.22				11.32 ± 9.09	11.98 ± 7.31			
Syringetins	6.24 ± 1.69	4.45 ± 1.22		*		2.16 ± 1.22	2.88 ± 1.08	**	*	
F3H-flavonols	4.78 ± 3.35	4.88 ± 3.22				11.32 ± 9.09	11.98 ± 7.31			
F3’5’H-flavonols	52.93 ± 9.69	41.47 ± 4.78		**		55.47 ± 5.16	57.11 ± 4.76	**		
F3’H-flavonols	42.29 ± 6.4	53.65 ± 7.43		**		33.21 ± 5.44	30.91 ± 3.36			
F3’H-flavan-3-ols	94.79 ± 0.78	93.01 ± 0.79		**		98.94 ± 0.13	98.38 ± 0.38		**	
F3’5’H-flavan-3-ols	5.21 ± 0.78	6.99 ± 0.79		**		1.06 ± 0.13	1.62 ± 0.39		**	
Catechin	3.13 ± 0.73	3.65 ± 1.1				30.68 ± 15.36	30.8 ± 9.87	**		
Epicatechin	89.97 ± 2.69	87.81 ± 3.21				66.3 ± 13.96	65.81 ± 8.48	**		
Gallocatechin	0.27 ± 0.16	0.49 ± 0.31	*	*		0.08 ± 0.01	0.14 ± 0.03		**	
Epigallocatechin	4.92 ± 0.71	6.47 ± 0.59		**		0.95 ± 0.13	1.44 ± 0.35		**	
Epicatechin gallate	1.69 ± 2.17	1.55 ± 2.13	**			1.96 ± 1.52	1.77 ± 1.35	**		
Epigallocatechin gallate	0.02 ± 0.02	0.03 ± 0.03	**			0.04 ± 0.02	0.04 ± 0.02	*		

* S, Skin; P, Pulp; C, Control; B, pbagging, *represents p < 0.05, **represents p < 0.01.

Values were reported as means ± SD. Significance results consisted of two-way ANOVA.

### 3.4 Screening for flavonoid biomarkers in Kolor skin and pulp with light-response differences

**Figure 4:**
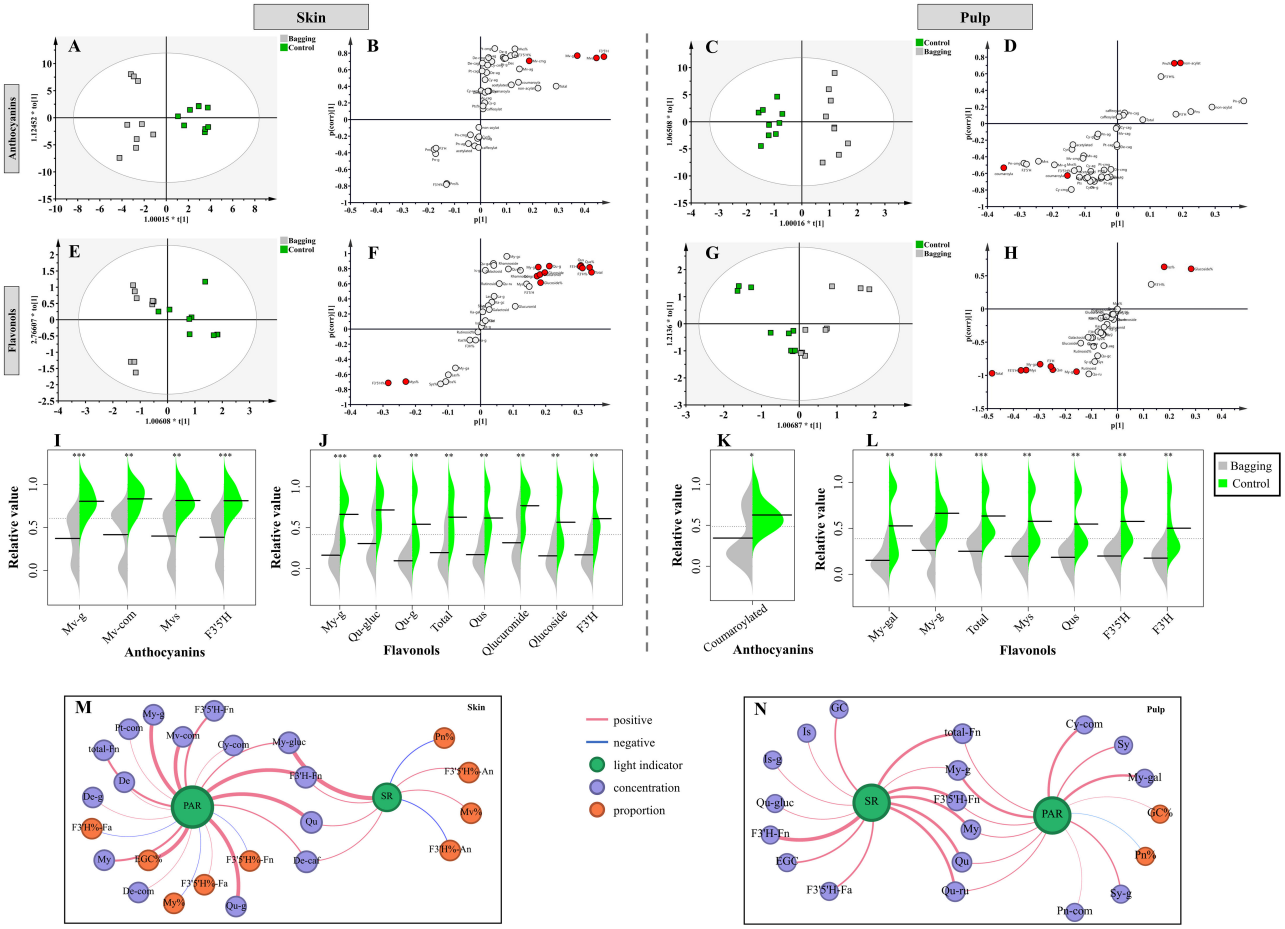
OPLS-DA plots **(A, C, E, G)**, S-plots **(B, D, F, H)** and relative value of biomarkers showing the separation of bagging treatment groups from control groups based on the concentrations and proportions of flavonoid compounds in berries. The results of skin **(I, G)** and the pulp **(K, L)** as shown. Network of light indicator and flavonoids in the skin **(M)** and pulp **(N)**. The blue line represents negative correlation and the red line represents positive correlation. The thickness of the line represents the weight of the correlation. (Cy, cyanidins; De, delphinidins; Pn, peonidins; Pt, petunidins; Mv, malvidins; My, myricetins; La, laricitrins; Qu, quercetins; Is, isorlrhamnetins; Ka, kaempferols; Sy, syringetins; An, anthocyanins; Fn, flavonols; Fa, flavan-3-ols; g, glucoside; com, coumarylated; caf, caffeoylated; gal, galactoside; gluc, glucuronide; ru, rutinosid; SR, solar radiation; PAR, photosynthetically active radiation).

To comprehensively investigate the impact of bagging treatment on flavonoids in Kolor skin and pulp, OPLS-DA was used to differentiate samples with distinct light-response differences, and biomarkers with VIP > 1 in the model were identified (|*p*| > 0.1, |*pcorr*| > 0.5). As illustrated in **Figures 4A-L**, bagging treatment influenced the levels of anthocyanins and flavonols in the skin and pulp of Kolor, grapes but had no significant impact on the levels of flavan-3-ols. In the context of the skin, two types of anthocyanins and three types of flavonols were selected. The concentrations of Mv-g and Mv-com significantly decreased in the skin following bagging treatment. The concentrations of My-g, Qu-gluc, and Qu-g, significantly decreased in the skin after shading. Additionally, there were notable reductions in the concentrations of Qu and the F3’H-flavonols. Bagging treatment had a discernible impact on the concentration of coumaroylated anthocyanins in the pulp of Kolor berries, with no significant differences in the concentrations of other anthocyanins. However, bagging treatment significantly increased the proportions of Pn and nonacylated anthocyanins. In the pulp, only My-gal and My-g exhibited differences in light response, with a significant decrease in concentration when occurring during bagging. The concentrations of My, Qu, and total flavonols all significantly decreased after bagging. Unlike those of anthocyanins, the concentrations of flavonols in the F3’5’H and F3’H pathways in the pulp also significantly decreased after treatment.

Correlation analysis was performed on two light indicators, PAR and SR, in relation to flavonoid compounds in both the skin and pulp of Kolor berries. **Figures 4M, N** demonstrate that flavonoids in the skin, which are correlated with both the SR and PAR, are predominantly composed of anthocyanins and flavonols. These substances exhibited a significant positive correlation with light intensity. Notably, anthocyanins in the skin such as De-g, Pt-com, Mv-com, Cy-com, De-com, and De-caf, exhibited a correlation of more than 80% with PAR, signifying a substantial reduction in their concentrations in bagged skin. Under these conditions, the correlations between the proportions of Mv, Qu and SR were 83% positive and 83% negative, respectively, which were consistent with the correlations between the proportions of F3’5’H- anthocyanins and F3’H-anthocyanins with the SR. These results suggested that under bagging conditions, the proportion of anthocyanins in the F3’5’H pathway in the skin significantly decreases, while the proportion of anthocyanins in the F3’H pathway increases. In Kolor skin, flavonols and PAR were strongly correlated, with My-g and Qu-g showing correlations of 98%, and the total flavonol content showing 87% correlation with PAR. The accumulation of these flavonols in the skin significantly decreased after shading. Moreover, the ratio of F3’5’H-flavonols to F3’H-flavan-3-ols in bagged berry skin increased, indicating a negative correlation with PAR. Bagging primarily affects flavonols in the pulp, and the concentrations of these compounds, including Is-g, Sy-g, My-g, My-gal, Qu-gluc, and Qu-ru, decreased with the shielding of SR and PAR during bagging. In the pulp, My, Qu, Is, Sy, F3’5’H- flavonols, F3’H-flavonols, and total-flavonols all exhibited a significant positive correlation with light intensity, indicating that the concentration decreased in the pulp of bagged berries. Consistent with the OPLS-DA results, the proportion of Pn in the pulp increased after bagging.

### 3.5 Effects of bagging treatment on the expression of genes associated with flavonoids in the skin and pulp of Kolor berries

**Figure 5:**
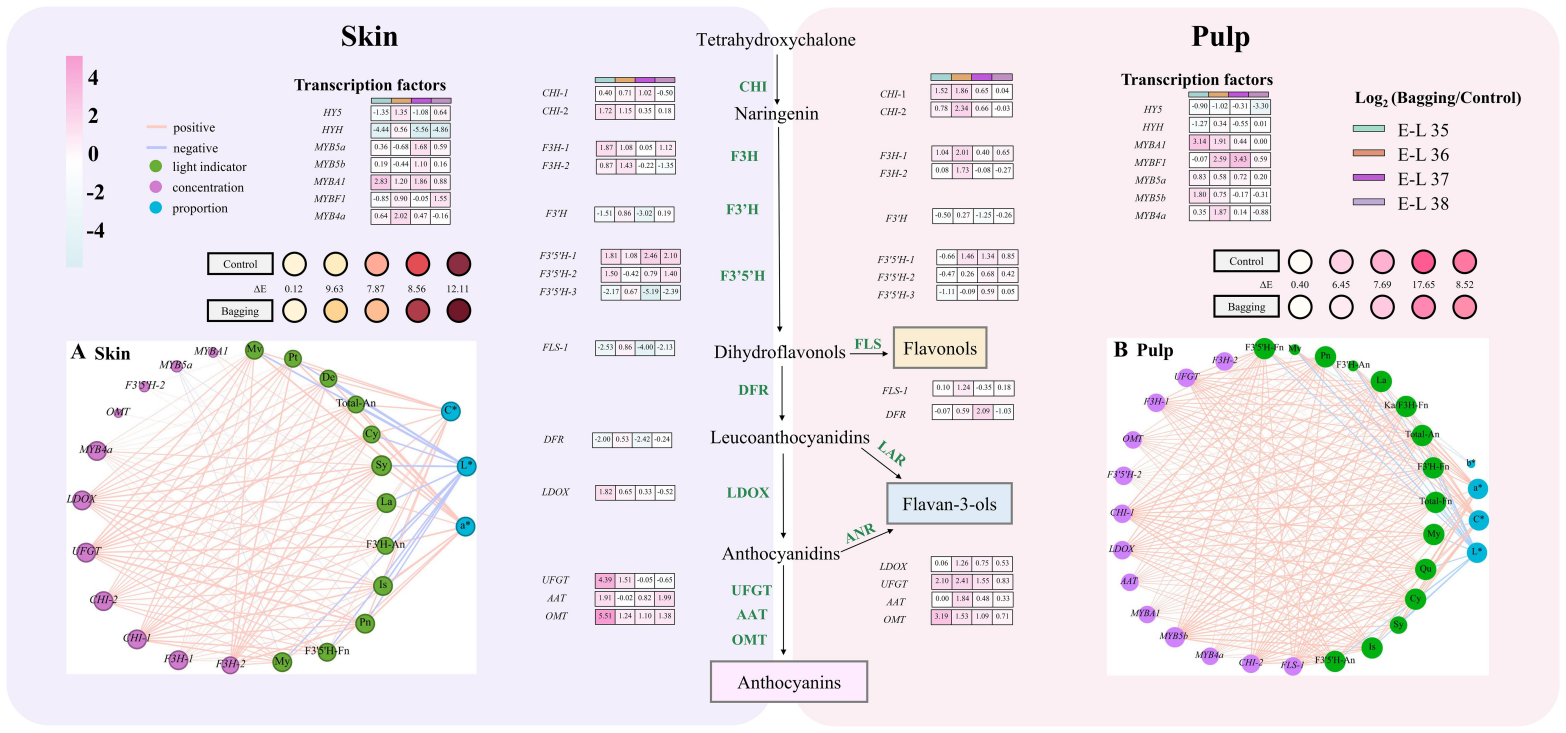
Flavonoid pathway related gene expression (log_2_[bagging/control]) patterns, and correlation network of flavonoids, gene expression and chromatic attributes in the skin **(A)** and pulp **(B)** in 2018. The left side is the results of skin and the right side is the pulp. The blue line represents negative correlation and the red line represents positive correlation. The thickness of the line represents the weight of the correlation. (Cy, cyanidins; De, delphinidins; Pn, peonidins; Pt, petunidins; Mv, malvidins; My, myricetins; La, laricitrins; Qu, quercetins; Is, isorlrhamnetins; Ka, kaempferols; Sy, syringetins; An, anthocyanins; Fn, flavonols).

Using RT-qPCR, the expression levels of crucial structural genes and transcription factors involved in the flavonoid synthesis pathway in the skin and pulp of Kolor berries were assessed in 2018. The expression levels of these 21 genes in the skin and pulp under different light conditions were monitored, as depicted in **Figure 5** and **Supplementary Figure 5**.

In Kolor skin, most structural genes exhibited high expression levels during the middle and later stages of veraison. Notably, the response of different genes to bagging treatment exhibited variability. After bagging, the expression of *UFGT* in Kolor grape skins was significantly downregulated from E-L 37 until maturity, similar to the expression patterns of *LDOX* and *F3H-2*. In the skin, the expression levels of *F3’5’H-3*, *FLS-1*, and *DFR*, except for a transient upregulation at E-L 36, decreased during other periods. The expression pattern of F3’H was similar to that of *F3’5’H-3*, but its expression level increased again during the mature stage. The expression patterns of *MYb5a* and *MYB5b* were opposite to those of structural genes such as *F3’5’H-3*. Following a decrease at E-L 36, the expression levels of these genes gradually increased during berry development. *MYb4a* maintained consistently higher expression levels than did the control group during veraison.

Conversely, the expression levels of *HY5* and *HYH* were notably lower in the pulp of Kolor grapes than in the skin. Following the implementation of a bagging treatment, the expression of *HY5* was downregulated, while that of *HYH* was initially upregulated at E-L 36, followed by subsequent downregulation at the E-L 35 and E-L 37 stages. Similarly, the expression levels of *HY5* and *HYH* in the pulp were notably lower than those in the skin, and their expression was downregulated following bagging treatment. The impact of shading treatment on *UFGT* in the pulp differed from its effect on the skin. *UFGT* expression in the pulp consistently exceeded that in the control group after bagging. The genes exhibiting comparable expression patterns included *LDOX*, *F3H-1*, and *CHI-1*. F3’H expression in the bagged pulp mirrored that in the skin. *F3’5’H-3* was downregulated before the completion of veraison. Subsequently, its expression gradually increased until berry maturation. The expression levels of *F3’5’H-1* and *F3’5’H-2* downregulated at the onset of veraison. Only *MYb5a* exhibited consistent upregulation in the bagged pulp, whereas *MYb4a* and *MYB5b* were significantly downregulated after veraison, indicating an opposite pattern to the changes in expression of *F3’5’Hs*.

A correlation analysis of the structural genes, and transcription factors associated with the flavonoid pathway and the flavonoid metabolites in the skin and pulp of Kolor berries, was conducted **Figures 5A, B** reveal strong correlations with anthocyanins and genes such as *LDOX*, *UFGT*, *CHI-1*, *CHI-2*, *F3H-1*, *F3H-2*, and *MYB4a*. The expression levels of these genes exhibited a significant positive correlation with the total-anthocyanins. In the skin, *FLS-1* was positively correlated with My, Is, and F3’5’H-flavonols, whereas *MYB5a* was negatively correlated with the accumulation of My, La, and F3’5’H-flavonols. In the pulp, anthocyanins were significantly and positively correlated with *F3H-1*, *F3H-2*, *LDOX*, *UFGT*, *AAT*, *MYB5b*, *CHI-1*, *CHI-2*, *MYBA1*, *MYB4a*, *OMT*, and *F3’5’H-2*. The flavonols in the pulp were positively correlated with *FLS-1*.

## 4 Discussion

### 4.1 Bagging altered the concentration and proportion of flavonoids in Kolor berries

Light, which encompasses light intensity, quality, and photoperiod, is the paramount environmental factor impacting flavonoid metabolism (Matus et al., 2009). In general, light promotes the production of flavonoids, notably anthocyanins and flavonols. In plants such as Arabidopsis and several others, anthocyanin synthesis is dependent on light exposure, as light exposure triggers the induction of anthocyanin biosynthesis-related genes and the accumulation of anthocyanins, ultimately resulting in grape colouration. Importantly, anthocyanins do not accumulate under conditions of low light or darkness (Maier and Hoecker, 2015). Zheng et al. (2013) revealed that Jingyan table grapes could accumulate a specific quantity of anthocyanins under shaded conditions, while Jingxiu grapes remained colourless. Red Globe (table grapes) is a classic light-dependent grape variety, in which the formation of skin pigments is driven by exposure to light (Sun et al., 2020). Excessive light and high temperatures typically adversely affect polyphenol accumulation in grape berries. Reducing light exposure appropriately benefits the accumulation of flavonoids in grape berries. In Mediterranean vineyards, the kaolin foliar application could enhance phenylpropanoid and flavonoid synthesis in grapes under conditions of elevated temperature and intense sunlight (Conde et al., 2016).

The Kolor grape variety employed in this study retained the ability to synthesize anthocyanins in both the skin and pulp even when subjected to shaded conditions. Although the synthesis of anthocyanins in Kolor berries is not contingent on light, the use of double-layer fruit bags for shading results in a noteworthy decrease in the overall anthocyanin content within the berries. This observation underscores the continued influence of light on the regulation of anthocyanin synthesis in Kolor grape plants. Environmental UV radiation is a potent inducer of polyphenol accumulation, with a notable emphasis on flavonols (Koyama et al., 2012). Specifically, UV-B radiation can increase the flavonol content in grape berries (Carbonell-Bejerano et al., 2014). Following the bagging process, the surfaces of Kolor berries were shielded from UV radiation, resulting in a significant reduction in the concentration of flavonols in the berries. This observation is consistent with earlier research findings. Fujita et al. (2006) reported that shading treatment significantly diminishes the content of native anthocyanins in grape skin, but has no impact on the native anthocyanin content in the seeds. Nevertheless, in our study, the concentration of flavan-3-ols in bagged Kolor berries during the ripening period exhibited no consistent pattern over three years. In addition to influencing the flavonoid concentration in grape berries, light conditions also assume a regulatory role in determining flavonoid proportions. Research indicates that varying light environments alter the ratio of dihydroxylated to trihydroxylated anthocyanins in grape berries, with reduced light elevating the concentration of nonacylated anthocyanins (Tarara et al., 2008). Comparable trends emerged in shading experiments involving teinturier grapes. The anthocyanin distribution in the F3’H pathway increased in both the skin and pulp of Yan 73 grapes, while the proportion of anthocyanins in the F3’5’H pathway decreased (Guan et al., 2014). Within Kolor berries, the shifts in anthocyanin proportions within the F3’H and F3’5’H pathways perfectly corroborate prior research outcomes.

### 4.2 Flavonoids in the skin and pulp of Kolor berries exhibited distinct responses to bagging

Kolor is a type of teinturier grape that accumulates anthocyanins in both the skin and pulp. There are differences in the concentration and proportion of anthocyanins between the skin and pulp, commonly referred to as tissue specificity. According to related studies on teinturier grapes, the concentration of anthocyanins in the skin is greater than that in the pulp of mature berries (Falginella et al., 2012). In our study of three consecutive years, we observed varying percentages of anthocyanins in the skin of Kolor berries—45%, 59%, and 47%. This finding contrasted with findings from previous studies (Falginella et al., 2012; Xie et al., 2015). Notably, during this study, in comparison to 2019, 2018 and 2020 experienced increased precipitation, coupled with reduced sunshine duration. It was postulated that the decrease in the anthocyanin content in the skin of Kolor berries during 2018 and 2020 in this study can be attributed to elevated rainfall and insufficient sunlight. The tissue specificity of teinturier grapes is evident in the concentration and proportion of anthocyanins, with the skin predominantly containing trihydroxy-substituted anthocyanins and the pulp primarily composed of dihydroxy-substituted anthocyanins (Xie et al., 2015; Guan et al., 2019). In the present study, 90%-94% of the trihydroxy-substituted anthocyanins in Kolor berries originated from the skin and accounted for only 12%-27% of the dihydroxy-substituted anthocyanins. Among them, Mv accounted for over 91% of trihydroxy-substituted anthocyanins in the skin, while Pn in the pulp constitutes more than 91% of the dihydroxy-substituted anthocyanins. Therefore, the tissue differences in anthocyanins in Kolor berries mainly depend on the disparity between Mv in the skin and Pn in the pulp. Following shading, the proportion of dihydroxy-substituted anthocyanins increased in both the skin and pulp of shaded grapes (Guan et al., 2016), consistent with our experimental findings. In the skin of bagged berries, trihydroxy-substituted anthocyanins decreased by 12%-26%, while dihydroxy-substituted anthocyanins increased by 61%-89%. These results suggested a divergence in light sensitivity between the two biosynthetic pathways responsible for synthesizing trihydroxy-substituted and dihydroxy-substituted anthocyanins. Flavonols in the Kolor berries can be classified into three pathways: the F3’5’H and F3’H pathways, along with the F3H pathway, which is mainly composed of kaempferol and its derivative compounds (Favre et al., 2018). However, the proportion of flavonols in this pathway did not vary notably in response to light. Qu and My are dihydroxy- and trihydroxy-hydroxy substituted flavonol compounds, respectively. The alterations in the proportions of quercetins and myricetins primarily caused the variations in the proportions of flavonols within the F3’H and F3’5’H pathways.

### 4.3 Differences in the light response of genes associated with the anthocyanin synthesis pathway in the skin and pulp of Kolor berries

The regulation of flavonoid metabolism in grapes can be divided into two primary categories: structural genes and regulatory genes. Research indicates that intense light fosters the accumulation of anthocyanins. This occurs as strong light augments the expression of transcription factors and structural genes within the anthocyanin biosynthesis pathway, while the expression of these genes markedly diminishes under conditions of low light or darkness (Jeong et al., 2004). In previous studies, it was observed that the pulp of red-fleshed grapes underwent colouration earlier than the skin did (Xie et al., 2015). Similar trends were noted in the investigation of Kolor berries. Following flowering, anthocyanins started accumulating in the pulp of Kolor berries, while the skin was not red. This observation further validated the tissue-specific expression of certain genes implicated in anthocyanin biosynthesis in Kolor berries.

The expression of the *CHS* gene is positively correlated with the increase in pigment content in berry skin (Harris et al., 2013). Research conducted in various plants, including *Arabidopsis*, mustard, and parsley, has demonstrated that the *CHS* promoter contains light-responsive elements such as ACE (ACGT-containing element) and MRE (MYB recognition element), leading to its induction by ultraviolet and blue light (Schulze-Lefert et al., 1989; Feinbaum et al., 1991; Kaiser et al., 1995). *LDOX* represents a downstream gene within the anthocyanin synthesis pathway. Gollop et al. (2001) reported that *LDOX* expression in grapes is initiated by exposure to light. An analysis of the promoter sequence revealed the presence of two Unit I light-responsive elements, facilitating the binding of *bZIP* and *MYB* transcription factors. *UFGT* assumes a pivotal role in grape skin anthocyanin synthesis, directly impacting grape skin colour based on its expression level. It is selectively expressed in the skin of coloured grape varieties during the ripening phase, and this expression is influenced by factors such as genotype, physiological state, and external environmental factors such as light and temperature (Bogs et al., 2005). *MYB4a* can suppress the expression of upstream genes within the phenylpropane biosynthesis pathway in grape berry skin (Cavallini et al., 2015). In the present study, the expression patterns of *F3H-2*, *LDOX*, *UFGT*, *CHI-1*, and *MYB4a* in the skin of bagged Kolor berries exhibited strong correlations with trends in anthocyanin and flavonol evolution. A decreased light intensity resulted in reduced expression levels of these genes, thereby governing the equilibrium of metabolic products and diminishing the synthesis of anthocyanins and flavonols in the skin. In this experiment, the expression of *MYB4a* decreased under shaded conditions. The concentration of anthocyanins in the Kolor pulp in 2018 did not decrease after shading. Therefore, downregulation of *MYB4a* expression reduced its inhibitory effect on the phenylalanine biosynthetic pathway, suggesting the possibility of synthesizing more anthocyanins under light exclusion conditions. In addition to those of the previously mentioned genes, the expression levels of *F3’H*, *F3’5’H-2*, and *MYB5b* also significantly correlated with the expression of flavonoid metabolites in bagged Kolor pulp. The expression of *F3’5’H* and *F3’H* played a pivotal role in shaping the fraction of anthocyanins. When the expression level of *F3’5’H* exceeded that of *F3’H*, the content of blue-purple products, predominantly composed of trihydroxy-substituted anthocyanins, increased. Conversely, the content of red products containing dihydroxy-substituted anthocyanins decreased (Jeong et al., 2006). Notably, *F3’5’H* found to be expressed predominantly in red grape varieties after the onset of veraison (Deluc et al., 2008). The tissue-specific accumulation of flavonoid compounds in the skin and pulp of teinturier grapes has been reported to be related to the expression levels of *VvF3’H* and *VvF3’5’H* (Mu et al., 2016). In this study (**Supplementary Figures 5E-H**), the expression of *F3’5’H* in the skin was significantly greater than that in the pulp, while the expression of *F3’H* in the skin and pulp was the same, except for a significant increase in expression in the E-L 37 stage in the skin. This was consistent with the findings of previous studies (Guan et al., 2014). However, in the present study, the expression levels of *F3’5’H-1* and *F3’5’H-2* in bagged Kolor skin and pulp significantly increased, revealing a pattern different from the changes in anthocyanin proportions. This may be due to the synthesis regulation of the anthocyanins and flavonols all by the upstream genes *F3’5’H* and *F3’H*. In Kolor berries, the inconsistency of the changes in the expression of *F3’5’H* and *F3’H* with previous reports may be due to variations in flavonol compounds, which exhibit showing opposite changes to those of anthocyanins (Downey et al., 2008). Interestingly, in previous studies, the expression level of *UFGT* in the pulp was lower than that in the skin, and shading decreased the expression level of *UFGT* (Xie et al., 2015; Guan et al., 2016). However, in the present study (**Supplementary Figure 5L**), the expression level of *UFGT* in the pulp of the control group was lower than that in the skin. After bagging, the expression level of *UFGT* in the pulp increased. This may also explain why the anthocyanin levels in the pulp did not decrease significantly after bagging.

Many studies have had revealed the involvement of various transcription factors in the regulation of grape flavonoid metabolism. Loyola et al. (2016) identified two bZIP family transcription factors, *HY5* and *HYH*, which collaboratively oversee the accumulation of grape flavonols and play a role in UV resistance. Notably, *HY5*, which encodes a bZIP-type transcription factor, was the pioneer gene discovered to be involved in promoting photomorphogenesis (Chattopadhyay et al., 1998). Azuma et al. (2015) conducted research demonstrating that the expression of *VvHY5* in grapes is initiated by light exposure, and that its transcript abundance varies across distinct stages of berry development. Subsequent investigations revealed two peaks in *VvHY5* expression during the development of Kyoho grapes, one aligning with the rapid accumulation of anthocyanins and the other occurring in the late phase of fruit ripening. These findings indicate that *VvHY5* expression is intricately tied to developmental progression and positively correlated with anthocyanin accumulation (Matus et al., 2017). These findings align with the observed expression pattern of *HY5* in Kolor skin during our experiment. When subjected to light exclusion conditions, both *HY5* and *HYH* experienced varying degrees of reduction in expression, and *HYH* was more sensitive to the absence of light (**Supplementary Figures 5T, U**), potentially impacting the expression of structural genes and transcription factors within the flavonoid pathway. This, in turn, indirectly leads to a decrease in the production of most flavonoid pathway metabolites.

Anthocyanins and flavonols serve as the primary pigments in grapes. Disparities in light exposure not only influence the inherent quality of grape berries but also affect their external colouration. Empirical findings suggest that dihydroxylated anthocyanins play a prominent role in grape varieties with lower overall anthocyanin contents, resulting in a red or pink hue in the skin. Conversely, trihydroxylated anthocyanins are frequently linked to darker-coloured grape varieties (Liang et al., 2011). After bagging, the concentration of dihydroxy-substituted anthocyanins in the skin increased, the a* value of the Kolor skin extract decreased, and the b* value increased. This pattern was also observed in the pulp. This study elucidated the flavonoid profiles of Kolor berries and screened out biomarker compounds sensitive to light exclusion conditions in the skin and pulp. The light sensitivity of genes related to the flavonoid biosynthetic pathway in Kolor skin and pulp was revealed. However, further research is needed to investigate the transcriptional regulatory mechanism of differential flavonoid synthesis in the skin and pulp.

## 5 Conclusion

Flavonoid compounds in grape berries showed variations at distinct developmental stages and under different light conditions. The concentrations and proportions of flavonoid compounds in the skin and pulp of teinturier grapes were similar, while tissue-specific variation was observed. Kolor berry skin accumulated abundant trihydroxy-substituted anthocyanins, while the pulp was rich in dihydroxy-substituted anthocyanins. Light intensity influenced flavonoids in different tissues, and the light-response of anthocyanins was also tissue-specific. Bagging increased the proportion of dihydroxylated anthocyanins in both the skin and pulp, but had no significant impact on the total anthocyanins in the pulp. The flavonols in the Kolor berries were more sensitive to light intensity, and decreased significantly after bagging both the skin and pulp. Malvidins and quercetins in the skin, and myricetins and quercetins in the pulp, were sensitive biomarkers of Kolor berries in response to light exclusion. After bagging treatment, the concentrations of these compounds significantly decreased. Bagging decreased the expression levels of *HY5* and *HYH* in both the skin and pulp, and the genes related to the anthocyanin synthesis pathway were downregulated after bagging. However, bagging did not downregulate the expression of *UFGT* in the pulp, resulting in a stable total anthocyanin content in the pulp. After shading, there were variations in the expression levels of the *F3’5’H*s and F3’H genes, and there were differences in the regulation of di-hydroxy and trihydroxy-substituted products of anthocyanins and flavonols.

## Data availability statement

The original contributions presented in the study are included in the article/**Supplementary Materials**, further inquiries can be directed to the corresponding author.

## Author contributions

HL: Conceptualization, Data curation, Formal analysis, Investigation, Software, Validation, Visualization, Writing – original draft, Writing – review & editing. XG: Writing – review & editing. YW: Writing – review & editing, Conceptualization, Validation. HL: Writing – review & editing, Validation. MT: Writing – review & editing, Validation. CD: Supervision, Writing – review & editing. JW: Conceptualization, Funding acquisition, Resources, Supervision, Writing – review & editing, Project administration, Validation.
