# Supplementary material for: Artificial shading of teinturier grape Kolor clusters reveals light-responsive biomarkers: focus on flavonoid profiles and metabolism
Source: Front Plant Sci. 2024 Mar 12;15:1356799. doi: 10.3389/fpls.2024.1356799 (PMC10963508; doi:10.3389/fpls.2024.1356799)
Supplement: Supplementary file 2 [file DataSheet_1.docx]

Supplementary Material

# Supplementary Figure 1


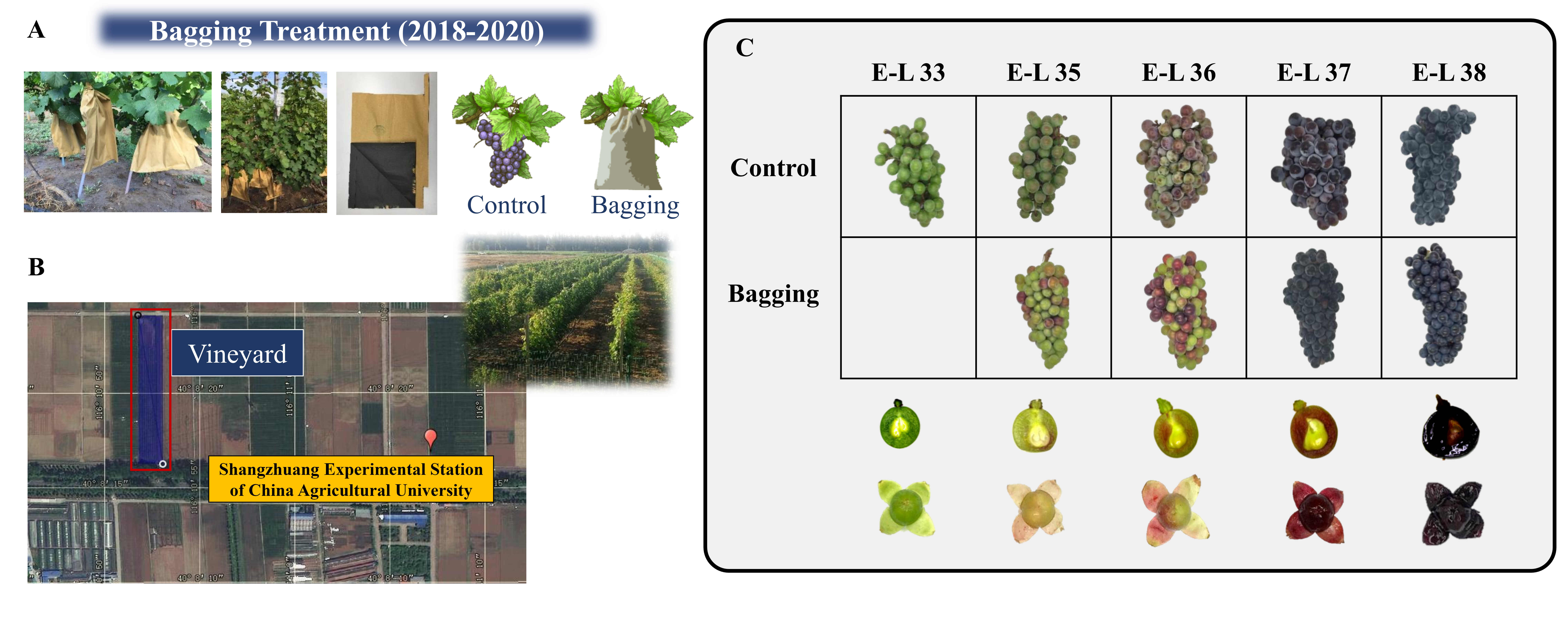


**Supplementary Figure 1** Fruit bags used in the experiment (A), location of the vineyard (B) and post-harvest photos of the grapes clusters and a cutaway view showing the Kolor berries at five developmental stages (C).

# Supplementary Figure 2


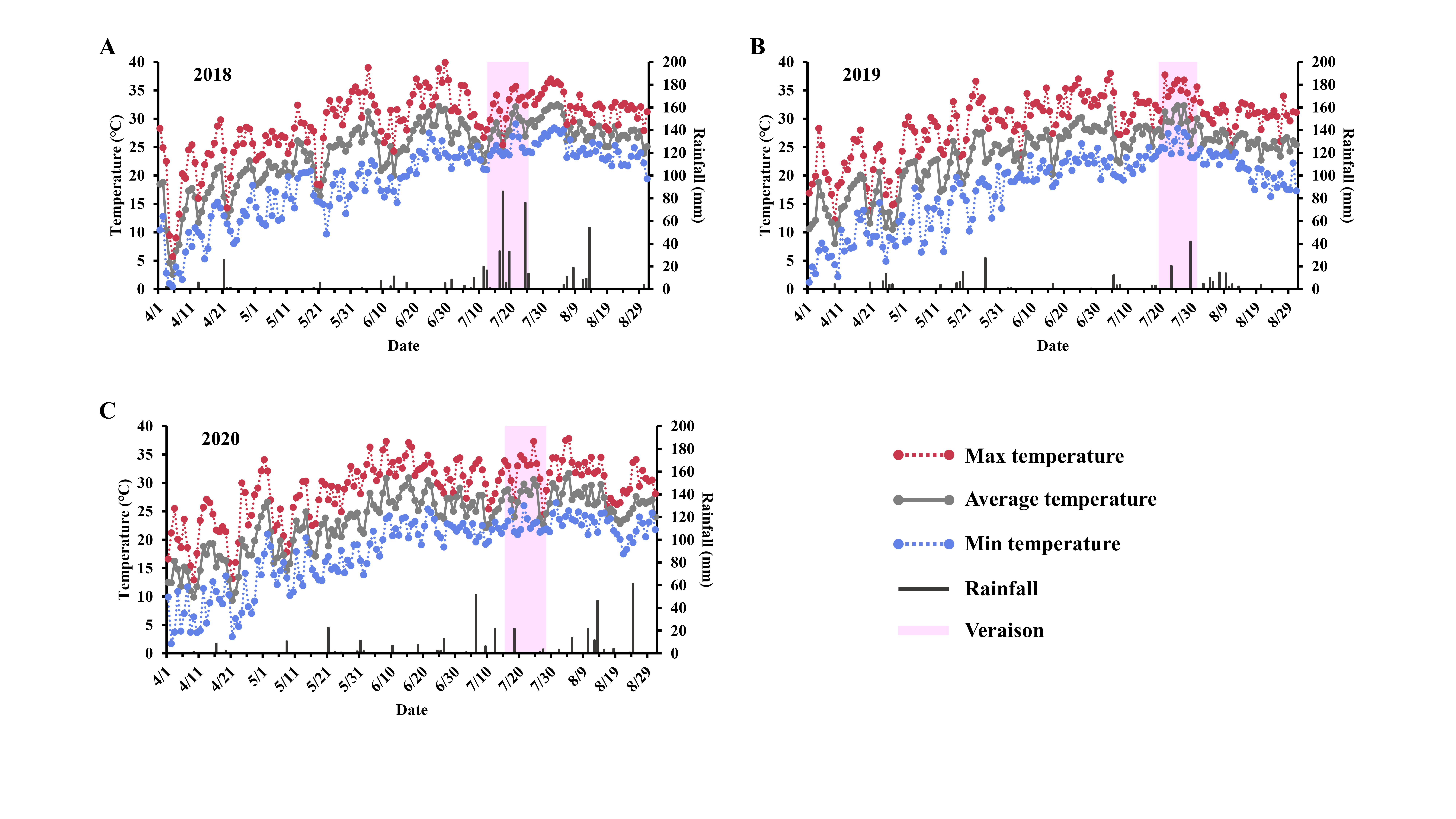


**Supplementary Figure 2** Meteorological data (maximum air temperature [red line], minimum air temperature [blue line], average air temperature [gray line], and precipitation [black bar]) of the experimental vineyard from April to August in 2018-2020. The veraison period was framed by pink area.

# Supplementary Figure 3


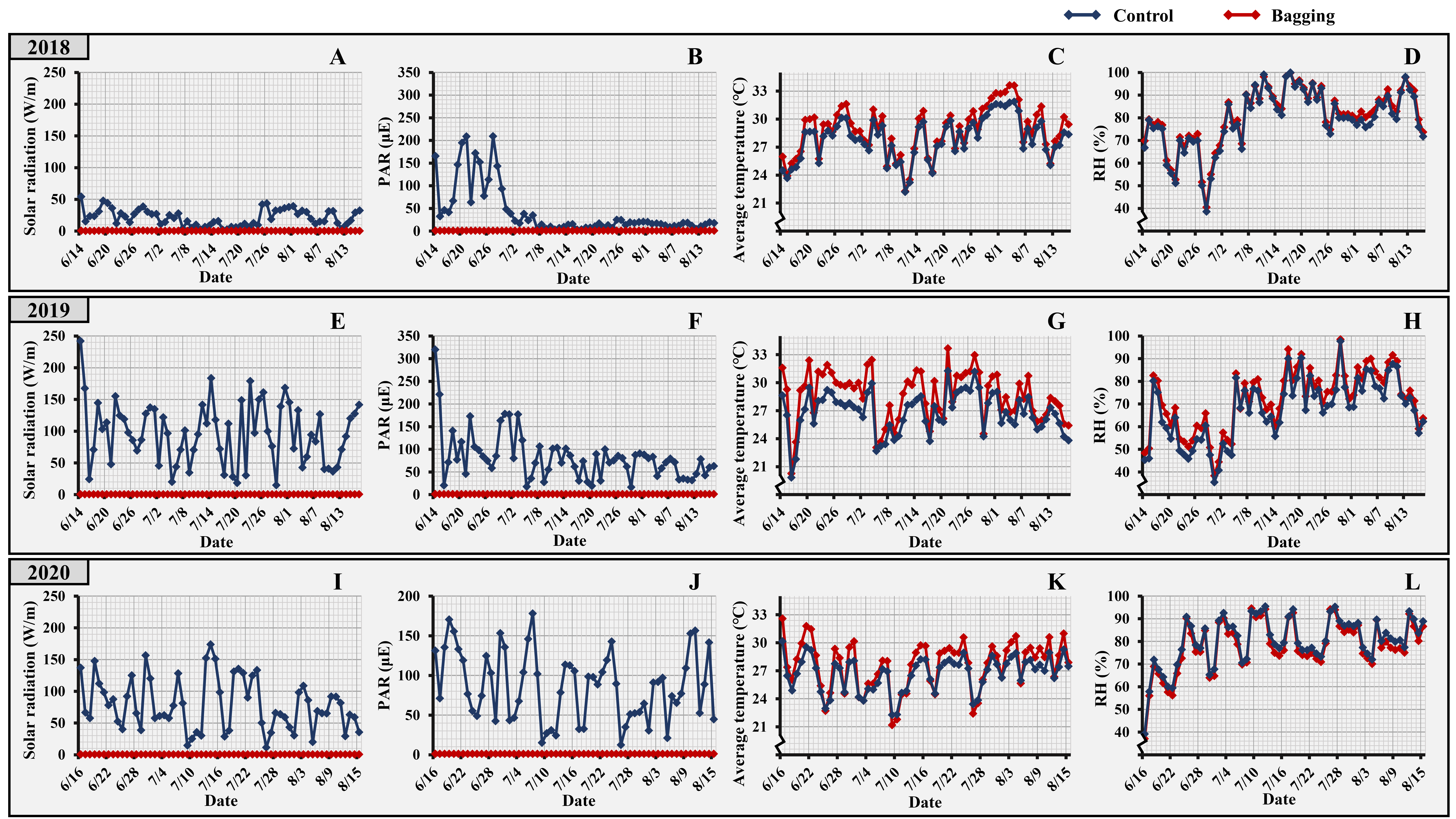


**Supplementary Figure 3** Microclimate changes of different treatment groups from E-L 33 to E-L 38. They are arranged solar radiation (A, E, I), PAR (B, F, J), average daily temperature (C, G, K), and relative humidity (D, H, L). The blue line represents the control groups and the red line represents the bagging treatment groups.

# Supplementary Figure 4


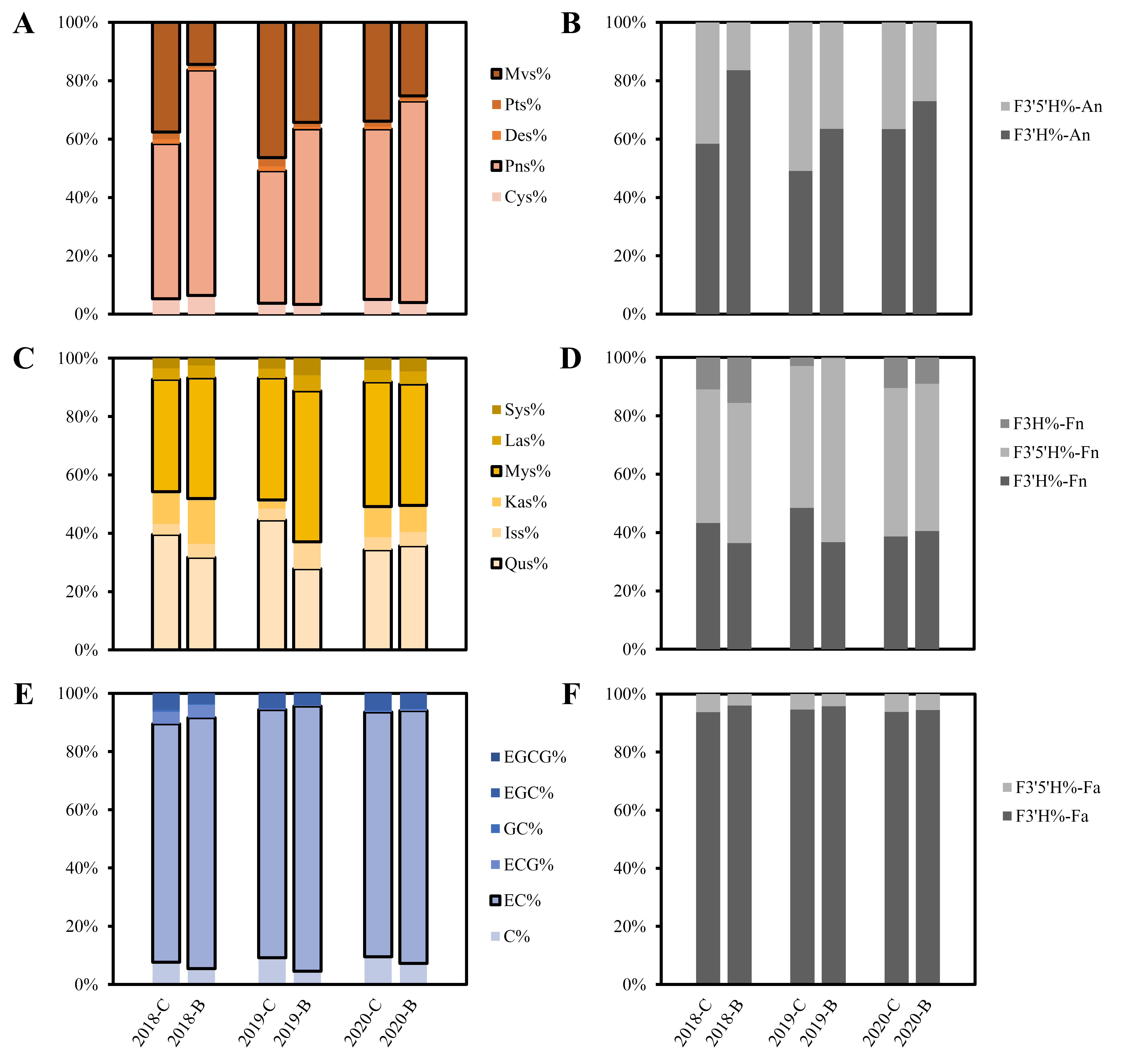


**Supplementary Figure 4** Proportion of anthocyanins (A, B), flavonols (C, D), and flavan-3-ols (E, F) in the Kolor berries (at E-L 38) under bagging treatment and control.

# Supplementary Figure 5





**Supplementary Figure 5** Relative normalized expression 14 structural genes (A-N), 5 *MYB* family transcription factors closely associated with flavonoid biosynthesis pathway (O-S), and 2 light-regulated factors (T, U) in the skin and pulp at five developmental stages. The dark blue line represents bagging groups and the yellow line represents control groups. * indicates there are signiﬁcant differences between bagging and control groups (*p* < 0.05, *t*-test).
